# Measuring the Refractive Index of Bovine Corneal Stromal Cells Using Quantitative Phase Imaging

**DOI:** 10.1016/j.bpj.2015.08.046

**Published:** 2015-10-20

**Authors:** Steven J. Gardner, Nick White, Julie Albon, Carlo Knupp, Christina S. Kamma-Lorger, Keith M. Meek

**Affiliations:** 1 Optometry and Vision Sciences, Cardiff University, Cardiff, UK

## Abstract

The cornea is the primary refractive lens in the eye and transmits >90% of incident visible light. It has been suggested that the development of postoperative corneal haze could be due to an increase in light scattering from activated corneal stromal cells. Quiescent keratocytes are thought to produce crystallins that match the refractive index of their cytoplasm to the surrounding extracellular material, reducing the amount of light scattering. To test this, we measured the refractive index (RI) of bovine corneal stromal cells, using quantitative phase imaging of live cells in vitro, together with confocal microscopy. The RI of quiescent keratocytes (*RI* = 1.381 ± 0.004) matched the surrounding matrix, thus supporting the hypothesis that keratocyte cytoplasm does not scatter light in the normal cornea. We also observed that the RI drops after keratocyte activation (*RI* = 1.365 ± 0.003), leading to a mismatch with the surrounding intercellular matrix. Theoretical scattering models showed that this mismatch would reduce light transmission in the cornea. We conclude that corneal transparency depends on the matching of refractive indices between quiescent keratocytes and the surrounding tissue, and that after surgery or wounding, the resulting RI mismatch between the activated cells and their surrounds significantly contributes to light scattering.

## Introduction

The cornea is the transparent window at the front of the eye that is responsible for approximately two-thirds of its refractive power. It consists of an outer epithelium, stroma, and an inner endothelium. The stroma makes up ∼90% of the tissue and comprises layers of collagen fibrils embedded in a matrix rich in hydrophilic proteoglycan molecules, together with flattened, quiescent cells called keratocytes. There is a mismatch in the refractive indices of the collagen fibrils and that of the rest of the extracellular matrix (1) such that, at a wavelength of 500 nm, ∼94% of the incident light should be scattered (2). From the seminal work of Maurice (2), subsequently refined by others (3, 4, 5), it is now generally accepted that transparency results from destructive interference of the scattered light in all directions except the forward one, brought about by the narrow, uniform size of the fibrils and the high degree of short-range order in their spatial arrangement (6).

Keratocytes are responsible for the maintenance of the collagen matrix that makes up the majority of the stroma, and are therefore essential for the continued transparency of the cornea. To date, the attempts to explain the observed transparency have so far focused only on the arrangement of the fibrils in the stroma and have ignored any contribution by stromal cells, even though these cells contribute up to 15% of the total stromal volume (7, 8). The justification for this was that these cells are very flat and thin in the direction of the passing light and that only the cell nuclei are observed to scatter light, with the cytoplasm remaining transparent. To explain these optical properties, Jester et al. (9), Jester (10), and Møller-Pedersen (11) hypothesized that corneal stromal cells produce crystallin molecules that remain in their cytoplasm, changing the refractive index to better match the surroundings and thus decrease scattering. This process is already well known to contribute to the transparency of the lens (3, 12, 13). In the mammalian cornea, the most likely candidate is a form of aldehyde dehydrogenase, ALDH3A1, which was first detected in the bovine cornea by Holt and Kinoshita (14), and later confirmed in two articles by Alexander et al. (15) and Silverman et al. (16). ALDH3A1 does not appear to catalyze any biological pathway in the cornea, although it has been shown to play a role in resistance to ultraviolet radiation (17) and oxidative stress (18, 19).

It is well known that when wounded, the corneal stroma scatters light at the site of the wound. During the healing process, keratocytes become activated to fibroblasts that migrate to the site of the damage, then proliferate, differentiate, and lay down fibrotic connective tissue. Studies of keratocyte responses to wounding, using confocal microscopic techniques, suggest that the activation of the keratocyte could be responsible for the increase in scattering (9). Any change in cytoplasmic content when the keratocytes undergo fibroblastic transformation is likely to alter the refractive index, which would then contribute to the increase in light scatter.

## Materials and Methods

### Refractive index measurements

To test the hypothesis that stromal cell differentiation is the main cause of scattering in a wounded cornea, a measurement of the refractive index of active and inactive stromal cells is needed. The refractive index (*n*) of any object can be calculated by measuring two other properties, the thickness (*t*) and the phase shift (Δ*Φ*) of light of a given wavelength (*λ*) that travels through the object, and using the relation between these values,

(1) Δϕ=knt,

where *k* = 2*π*/*λ* and assuming negligible refraction at the surface. When moving from one medium of refractive index *n*_1_ to another of refractive index *n*_2_, this relation becomes

(2) $$\text{Δ}\phi =kt\left(n_{2}-n_{1}\right).$$

Thus, to measure refractive index, it is necessary to measure both the thickness of the object in question and the phase shift of the incident light of a known wavelength.

### Background to quantitative phase imaging

The phase shift caused by the cells in vitro can be measured by using the technique of Paganin and Nugent (20), who produced phase images by solving the transport-of-intensity equation explicitly for phase. The transport of intensity equation is derived under the assumption that the amplitude of the propagating light satisfies the parabolic equation (21). Once applied to light traveling along the *z* direction, and after some manipulation, the parabolic equation reduces to the transport-of-intensity equation

(3) $$-k\partial_{z}I\left(\vec{r}\right)=\nabla .\left[I\left(\vec{r}\right)\nabla \phi \left(\vec{r}\right)\right],$$

where *I* is the irradiance and *φ* is the phase of the incoming light. The value ∂_*z*_ is the partial differential operator with respect to the *z* coordinate, ∇ is the two-dimensional partial differential operator with respect to the *x* and *y* coordinates, and *k* = 2*π*/*λ* as before. A complete derivation of this result can be found in Teague (21). Throughout this article, a Cartesian coordinate system will be used where *x* and *y* represent positions within the plane of the image, and *z* is the axis perpendicular to that plane. If we assume that the irradiance is uniform within a plane (*I*_0_), Eq. 3 can be rearranged to

(4) $$\nabla^{2}\phi \left(\vec{r}\right)=\frac{-k}{I_{0}}.\frac{\partial I\left(\vec{r}\right)}{\partial z},$$

allowing the phase to be explicitly calculated by measuring the irradiance at different *z* positions, assuming that the transport-of-intensity equation is generalized for partially coherent light (20) and thus that *k* is a constant. This technique has been used by Curl et al. (22) to measure the refractive index of airway smooth muscle cells. A broadly similar method to that of Curl et al. (22) is used here.

### Scattering modeling

Once the refractive index and the cell size are known, the expected amount of scattering that a mismatch would cause can be calculated, using a simplified model. Because the cells are much larger than the wavelength of the incident light, we must use the established theory of Mie scattering (23). However, in its pure analytical form, Mie scattering is only applicable to spherical particles. A spherical cell model would not be suitable, as stromal cells in vivo lie flat between collagen lamellae, with a reasonably constant anterior to posterior thickness (24). To apply Mie scattering theory to shapes with less symmetry, the anomalous diffraction theory (ADT) of van de Hulst (25) can be used to simplify the calculation of the scattering cross section, while only losing information from secondary and tertiary structure. ADT was originally applied to spheres in a system where the wavelength is much smaller than the diameter of the particles and the ratio of refractive indices inside and outside the particle is close to 1. van de Hulst (25) showed that the effect of loss of information has little effect on the magnitude of the scattering cross section even for refractive index values as high as 1.7. Napper (26) was the first to apply ADT to cubic structures, in various orientations, and we can use this established technique to model stromal cells as flat cuboidal slabs. Following the argument of Napper (26), starting from the definition of scattering cross section as the ratio of total flux scattered per unit flux, it was shown (26) that for a face incident cube of cross-sectional area *A*, the scattering cross section, *σ*_*c*_, is given by

(5) $$\sigma_{c}=2A\left(1-\text{cos}\;\delta_{c}\right),$$

where *δ*_*c*_ = 4*πl*(*m*−1)/*λ*, the phase shift of light of wavelength *λ* caused by a refractive index ratio of *m* along a path of length *l*.

Once the scattering cross-section has been calculated, we can calculate the transmission through the whole of the cornea using the relation

(6) $$T=I/I_{0}=\text{exp}\left(-\varphi_{c}\sigma_{c}l/V_{c}\right)$$

for a cornea of thickness *l* and volume fraction of *φ*_*c*_ cells of volume *V*_*c*_. An interesting result of this for cuboidal cells is that the transmission through the cuboid is not dependent on the cross-sectional area. This can be clearly seen by substituting Eq. 5 into Eq. 6,

(7) $$T=\text{exp}\left(-\varphi_{c}\sigma_{c}l/V_{c}\right)=\text{exp}\left(-2\varphi_{c}l\left(1-\text{cos}\;\delta_{c}\right)/t_{c}\right),$$

where the thickness of the cell is *t*_*c*_ = *V*_*c*_/*A*.

### QPI validation

**Figure 1:**
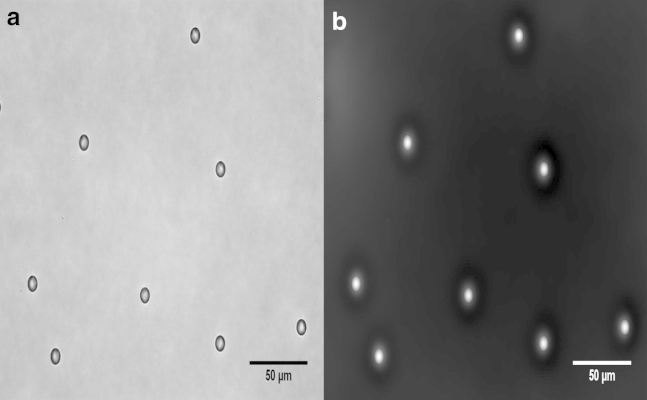
Central focal plane bright-field (*a*) and phase (*b*) images of polystyrene beads of size 10.00 ± 0.04 *μ*m.

The method of phase measurement was first tested using traceable polystyrene microspheres (National Institute of Science and Technology (NIST), Gaithersburg, MD)) of a known refractive index (1.59 at 589 nm) obtained from Thermo Scientific (Fremont, CA). Spheres, of size 10.00 ± 0.04 *μ*m and 15.02 ± 0.08 *μ*m (mean ± SD), were suspended in distilled water and imaged under regular bright-field transmitted light. Köhler illumination conditions were used to satisfy the uniform irradiance assumption of Eq. 4, and a 540-nm green filter of a full width at half-maximum bandwidth of 90 nm was used to provide an accurate central wavelength. Image stacks of increasing *z* through the focal plane were obtained using a model No. DM6000B upright microscope at 1-*μ*m intervals for a total distance of 30 *μ*m and a 20× Plan-Apochromat air lens (Leica Microsystems, Wetzlar, Germany) with a numerical aperture of 0.7, combined with a 1.6× additional magnification and a 0.7× camera mount. The charge-coupled device camera (Leica Microsystems) had a pixel size of 6.45 × 6.45 *μ*m. The best focal plane was set at the center of the bead by visual inspection by minimizing the out-of-focus diffraction rings arising from the bead/medium boundary. Images were acquired as a through-focal series either side of this plane. The focal image, combined with two out-of-focus images 2 *μ*m in the *z* direction from the focal plane, were imported into QPm software obtained from Iatia (Box Hill North, Victoria, Australia). The three images were used by the software to measure the irradiance gradient $\left(\partial_{z}I\left(\vec{r}\right)\right)$, and after image transforms were conducted according to Eq. 4, the software returned a quantitative phase image (QPI). Measurements were taken from pixels in straight lines across the center of each bead at different angles. These values were then combined with the known thickness of the sphere as a function of the distance from the center to determine the refractive index as per Eq. 2. The thickness of the sphere was calculated using the equation of a circle (a slice through the center of the sphere), which can be rearranged to give

(8) $$t_{s}=2{\left(r^{2}-a^{2}\right)}^{\frac{1}{2}},$$

where *t*_*s*_ is the vertical thickness of the sphere of radius *r* at a distance *a* from the central vertical axis. The central pixel of each bead was taken to be where the maximum phase shift was recorded. Phase-shift measurements near the edge of the sphere were discounted as the lensing effect was most pronounced at the sharp edge of the bead, which cannot be fully resolved. This effect manifested in the bright-field images as dark rings of no signal around the edge of the beads, which can be seen in Fig. 1 *a*.

NIST traceable polystyrene beads were measured to have a refractive index of 1.591 ± 0.004 (standard deviation, *n* = 74). This matches the manufacturer’s quote of 1.59 (no error was supplied). Fig. 1 shows an example of the focal plane image and the phase image that was produced, with the phase image being scaled such that the maximum phase shift is represented by the largest grayscale pixel value (*white*).

### Cell culture of activated bovine fibroblasts

Bovine eyes were obtained from a local abattoir. After removal of extraneous muscle and fat tissue, eyes were washed in 0.9% saline before immersion in 5% betadine solution for 5 min; eyes were again washed in saline. After corneo-scleral disk excision, the epithelium and endothelium were removed by scraping; then the stroma was cut into tissue explants. Stromal tissue was then placed in multiwell plates with DMEM (Dulbecco’s modified Eagle’s medium, without phenol red indicator) containing 10% FBS (fetal bovine serum) to stimulate cell growth. The tissue explants were cultured at 37°C, in an atmosphere of 5% CO_2_ until the activated stromal cells migrated out of the tissue. The tissue explants were then removed and the remaining cells immediately trypsinized (1× TrypLE Express; Invitrogen, Paisley, UK). The cell suspension was twice centrifuged at 3000 rpm for 8 min, with cell pellets being resuspended in DMEM each time. The final resuspended pellet was transferred to a 25 cm^3^ culture flask. After 2–3 days, when the cells attained confluence they were trypsinized again and split into additional flasks or seeded onto glass-bottomed petri dishes (MatTek, Ashland, MA) ready for image collection. Cells were fed with DMEM every 48 h.

### Cell culture of bovine keratocytes

Bovine eyes were cleaned, immersed in betadine and corneo-scleral discs were excised as described above. After removal of endothelial and epithelial cells (see above), stromal tissue was cut into ∼1 mm^3^ sections. These sections were incubated at 37°C in minimum essential medium containing 2 mg/mL collagenase type I. After 2 h, any remaining large pieces of stromal tissue were removed from the tube and the remaining solution was centrifuged at 500 rpm for 5 min. The resultant cell pellet was resuspended in 1 mL of DMEM solution with added nonessential amino acids, RPMI vitamin mixture, glutathione, a 1% v/v penicillin and streptomycin solution, and 0.1% v/v fungizone (amphotericin-B), seeded onto culture flasks and cultured at 37°C in an atmosphere containing 5% CO_2_. After cell adherence to the flask, further culture medium was added. When the cells had reached confluence, they were imaged under bright-field conditions, while still being kept in an incubated environment.

### QPI collection

The QPIs of cells were acquired in the same way as described for the bead images. Wide-field, transmitted light, bright-field contrast image stacks were obtained at 1-*μ*m intervals in the *z* direction using a model No. DM6000 B microscope equipped with a HC Plan Apochromat 20× lens (0.7 numerical aperture; Leica Microsystems). Phase images were produced using the QPm software (Iatia), as with the beads. Pixel values were exported from the data contained within the phase image from 15 × 15 boxes placed in regions of the cell with the greatest phase retardation, rather than in lines, as this was assumed to be the area of greatest thickness. Mean values of phase retardation were calculated and used to calculate the refractive index, using Eq. 2.

### Confocal image collection

Unlike in the case of the beads, the thickness of the cultured cells is unknown, and as such must be measured for the refractive index to be calculated. To make this measurement, FITC (fluorescent isothiocyanate) dextran (average molecular weight 10,000, Stokes’ radius 2.3 nm) was added to the dish containing the cells and DMEM medium. The cells were then imaged under confocal fluorescence microscopy using 488-nm Argon laser illumination. In theory, because the dextran molecules were too large to penetrate the cell membrane, this would create images of fluorescent background with void areas where the cells were located. In practice this was mostly true, with most of the cells successfully blocking the intrusion of the fluorescent dye. The few cells that appeared to have the dextran leak into them were not used for the purposes of measurements.

Image stacks in the *z* direction were then acquired from immediately beneath the coverslip to the point at which the void disappeared. Images were captured using a 20× air lens with a numerical aperture of 0.8, at 0.05-*μ*m intervals using a model No. LSM5 Pascal confocal microscope and Axiovert 200 inverted stand (Carl Zeiss, Jena, Germany). The image stack was analyzed by creating intensity profiles within and adjacent to the void, and the upper cell boundary was taken to be where the intensity returned to 50% of the background level. Stacks were taken in periods of no more than 1 h, to prevent, as much as possible, changes in temperature from affecting the morphology of the cells. The resolution of the height data was taken to be one full focal step, which in this case was 0.05 *μ*m.

### Scattering modeling

**Figure 2:**
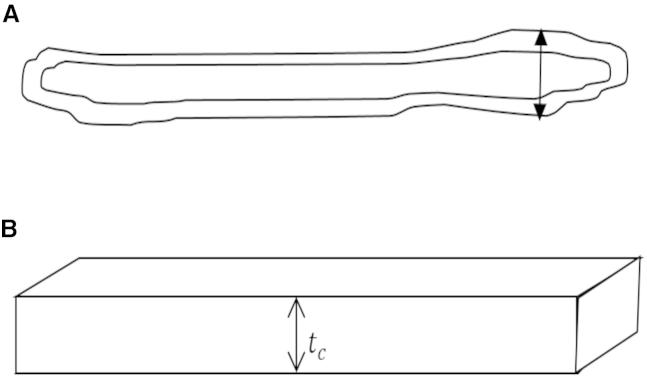
Schematic diagram to show transverse section of a typical corneal cell in vivo (*A*) and the particulate shape used in the scattering model (*B*). (*Arrow* in *A*) Point of maximum thickness, which was used as the basis of *t*_*c*_.

Corneal cells (keratocytes and fibroblasts) were modeled as cuboidal slabs of constant anterior-posterior thickness and constant refractive index, surrounded by extracellular material of refractive index 1.375 (2, 27) (Fig. 2). Transmission through a human cornea of thickness 550 *μ*m, as reported in Ambrósio et al. (28) and Cerviño et al. (29), was calculated according to Eq. 7. Two different cell thicknesses were used to represent the upper and lower limit of the size of a given cell type in vivo. Thickness measurements of ∼1 *μ*m reported from electron microscopy studies (24, 30) were considered to represent the lower limit, as although the micrographs represent the cells in a more natural environment, the processing required to produce them, in particular the dehydration and fixation stages, is known to cause tissue shrinkage. Although steps were taken to minimize the possible effects of shadow bleeding during the thickness measurements of the cultured cells, they may still contribute to an increased thickness measurement. As such, the thickness reported from results of confocal imaging were considered, for the purposes of the modeling work, to be an upper limit on the thickness.

## Results

**Figure 3:**
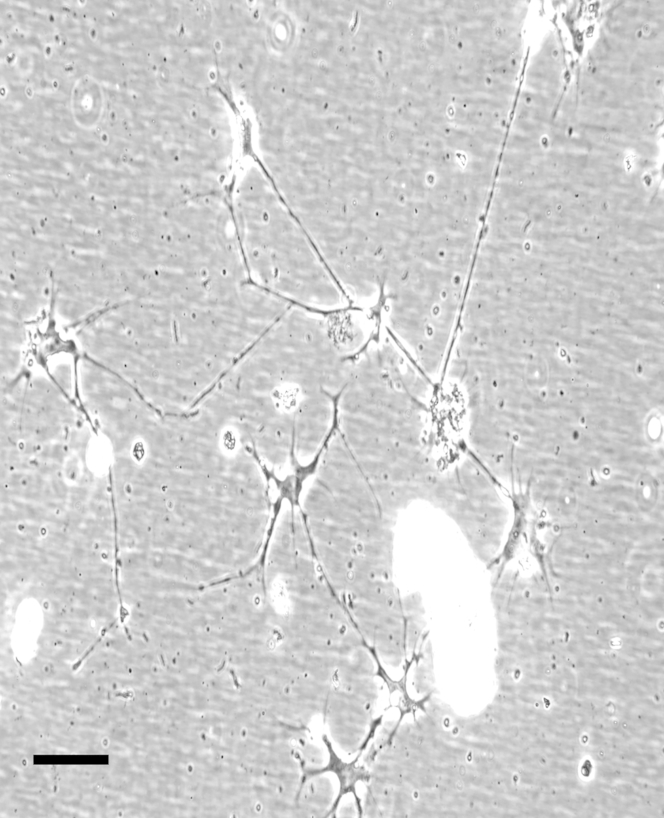
Phase contrast images of keratocytes cultured in serum free media. Scale bar = 25 *μ*m.

Under many culture conditions, stromal keratocytes become activated to fibroblasts. To check that the serum-free culture conditions we used had avoided this transformation, we imaged the keratocytes using phase contrast (Fig. 3). Cell morphology in the images, together with the characteristic presence of numerous cell processes, confirmed their status as keratocytes.

**Figure 4:**
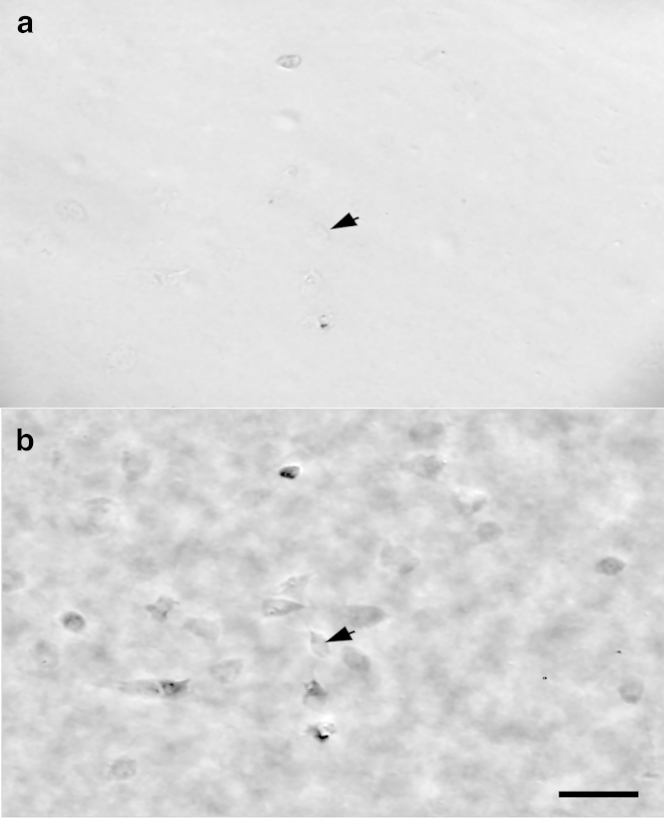
Bright-field (*a*) and computed QPI phase (*b*) images for keratocytes. (*Arrows*) Equivalent places between bright-field and phase images. Bar = 100 *μ*m.

Bright-field and QPIs of these keratocytes are shown in Fig. 4. The QPIs for cells are displayed with reversed contrast compared to that of beads (Fig. 1), to improve visualization. It should be noted therefore, that darker areas in the phase image of the cell indicate a greater phase shift.

**Figure 5:**
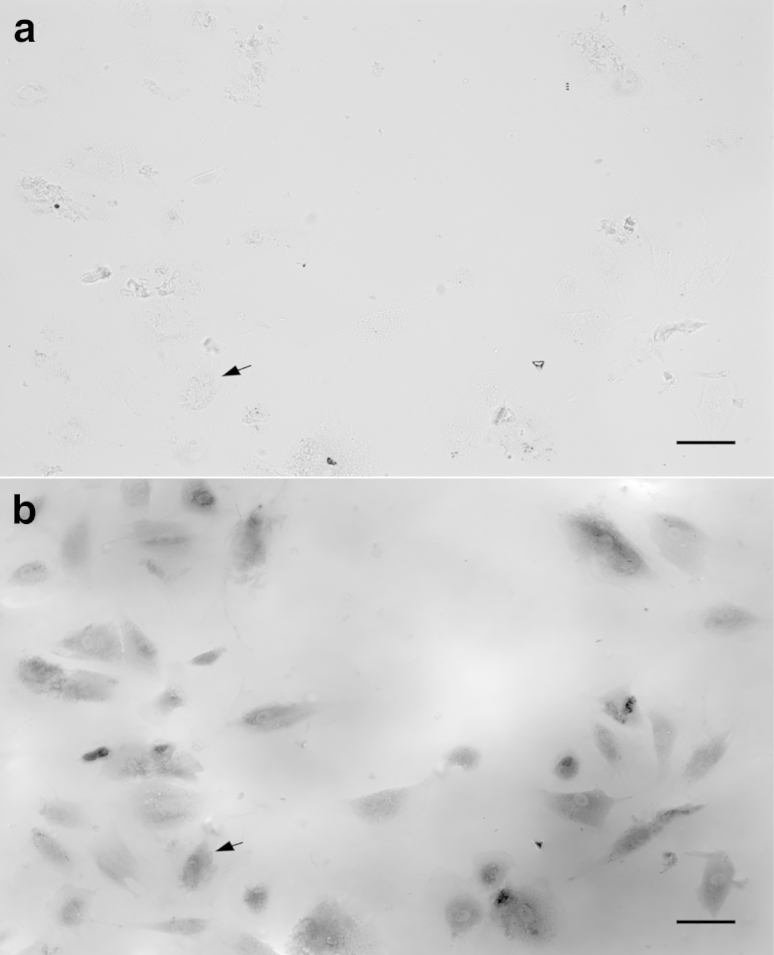
Bright-field (*a*) and computed QPI phase (*b*) images for fibroblasts. (*Arrows*) Equivalent places between bright-field and phase images. Bar = 50 *μ*m.

Fig. 5 *a* shows an acquired bright-field image at optimal focus for activated stromal fibroblasts. Fig. 5 *b* shows the computed QPI for the same cells.

Results for bovine cells are summarized in Table 1. Values of refractive index (*n*) were calculated using Eq. 2, with the refractive index of the medium taken to be 1.337 (31). Uncertainty values are the standard error for phase shift and refractive index measurements, and standard deviation for thickness. This is because the thickness was measured once for every cell (*n*), but the phase was measured 225 times (i.e., at every pixel in a 15 pixel square) for each cell, and was therefore a mean of sample means. The results demonstrate that the refractive index of the cells decreases significantly when the cells are activated (*p* < 0.0004, *t*-test with unequal variance).

**Figure 6:**
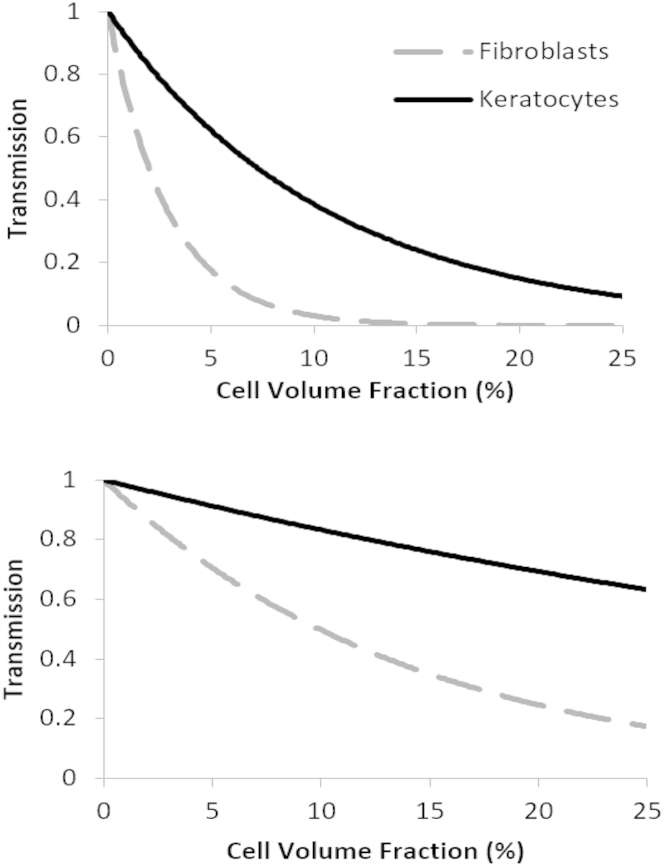
Plots showing transmission through a cornea of thickness 0.55 mm for increasing volume fraction of keratocytes and fibroblasts, for cell thicknesses from Table 1 (*top*), representing maximum values, and for mean cell thickness of 1.34 *μ*m for keratocytes (24), and of 1.82 *μ*m for fibroblasts (32) (*bottom*), representing minimum values.

Assuming that the refractive index of corneal keratocytes and fibroblasts is not species-dependent, we used these values together with the thickness of the human cornea (0.55 mm) to predict light transmission through the human cornea populated uniformly by keratocytes or fibroblasts as a function of cell volume fraction (Fig. 6). The predicted transmission through a cornea comprising inactive cells remains higher at normal volume fraction concentrations, which are known to range from 3 to 5% in the posterior stroma up to 12% just below Bowman’s layer (33). Upon activation, a significant drop in transmission is predicted for all cell concentrations.

## Discussion

The refractive indices reported here have been obtained under a green light filter, and so the specific refractive indices quoted here can only be confidently used under the same light conditions. There will be some wavelength dependence of refractive index, but the changes should be small across the rest of the visible band.

**Figure 7:**
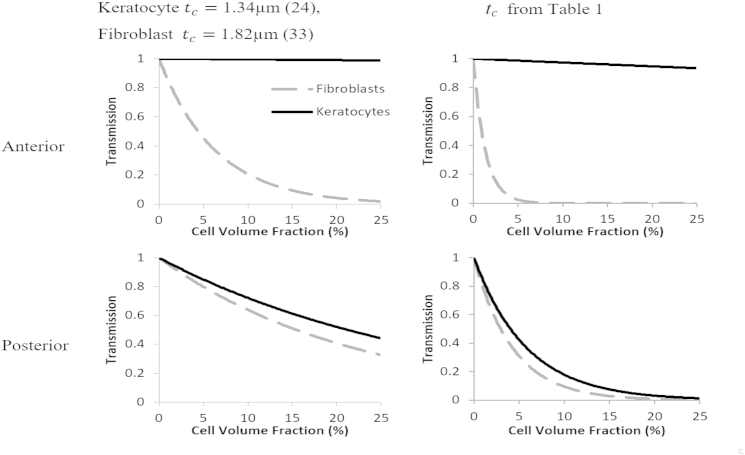
Plots to show theoretical transmissions through a cornea of 0.55 mm thickness using the upper (anterior stroma, *top panels*) and lower (posterior stroma, *bottom panels*) limits of the refractive index gradient reported by Patel et al. (39). As before, graphs are presented for corneas populated by keratocytes or fibroblasts of refractive index presented in Table 1 (*right panel*), and of lower limits on cell thickness (*left panel*).

It may be natural to assume that the small change in refractive index noted in Table 1 could not account for the dramatic drop in transparency that has been observed in previous experimental studies. However, even though this difference is small, the results from the theoretical model show that, if the volume fraction of cells were >10% as is observed in the anterior section of the cornea, then a change of this magnitude alone could cause the increase in scattering measured by Jester et al. (9), as well as that observed in postoperative case reports (34, 35) and animal models. At concentrations of ∼5% in the posterior section, the calculated 70% transmission for a cornea populated by activated fibroblasts shows that the cells would still be a major factor in the observed haze, even if they could not account for total opacity. If, however, we also take into account the increase in the cell density at the wound site, due to both the mobility of the newly activated fibroblasts and their propensity for mitosis during the wound-healing response, the opacity could be fully explained. This would also account for the reduction in light scattering after the cells either disperse or undergo apoptosis on completion of the wound-healing process. The findings of Jester et al. (9) showed that the increase in scattering is most pronounced in the anterior stroma, and this is consistent with our results given that the anterior section is that which has the highest keratocyte density (33, 36, 37, 38) and the highest refractive index mismatch (39). The value of refractive index of the extracellular material in the cornea of 1.375 is a mean value, and a large variability could have catastrophic effects on the clarity of the cornea even if the mean remains at a reasonable value. The refractive index gradient measured by Patel et al. (39) showed that the stromal refractive index decreases through the cornea from 1.380 ± 0.005 in the anterior section to a minimum of 1.373 ± 0.001 in the posterior region, thus giving a maximum refractive index mismatch of 0.008 for keratocytes. However, because this is in the posterior region where the cell volume fraction is much lower, the transmission remains relatively unaffected for cell thicknesses reported from electron microscopy studies (24, 30). The larger predicted change in transmission for the thicknesses measured here (Fig. 6) suggests that the thickness measurement in vitro does not describe the system in vivo, although it should be noted that the plot shows the consequence of a stromal refractive index of 1.375 (close to the minimum refractive index measured by Patel et al. (39)) being representative of the full thickness of the cornea, and not just of the posterior section. In addition, the thickness measurement of the cells could have been overestimated by the lensing effect of the cells themselves. As such this should be considered as an absolute lower limit for transmission though a healthy cornea populated with quiescent cells. We can extrapolate our results to consider the effects of this refractive index gradient. For fibroblasts the maximum refractive index mismatch is 0.015, and considering that this is in the anterior section where the cells are most concentrated, this would cause a much larger change in the transmission (Fig. 7). This is again consistent with the findings of Jester et al. (9) that scattering increases are more pronounced in the anterior section of the stroma.

**Figure 8:**
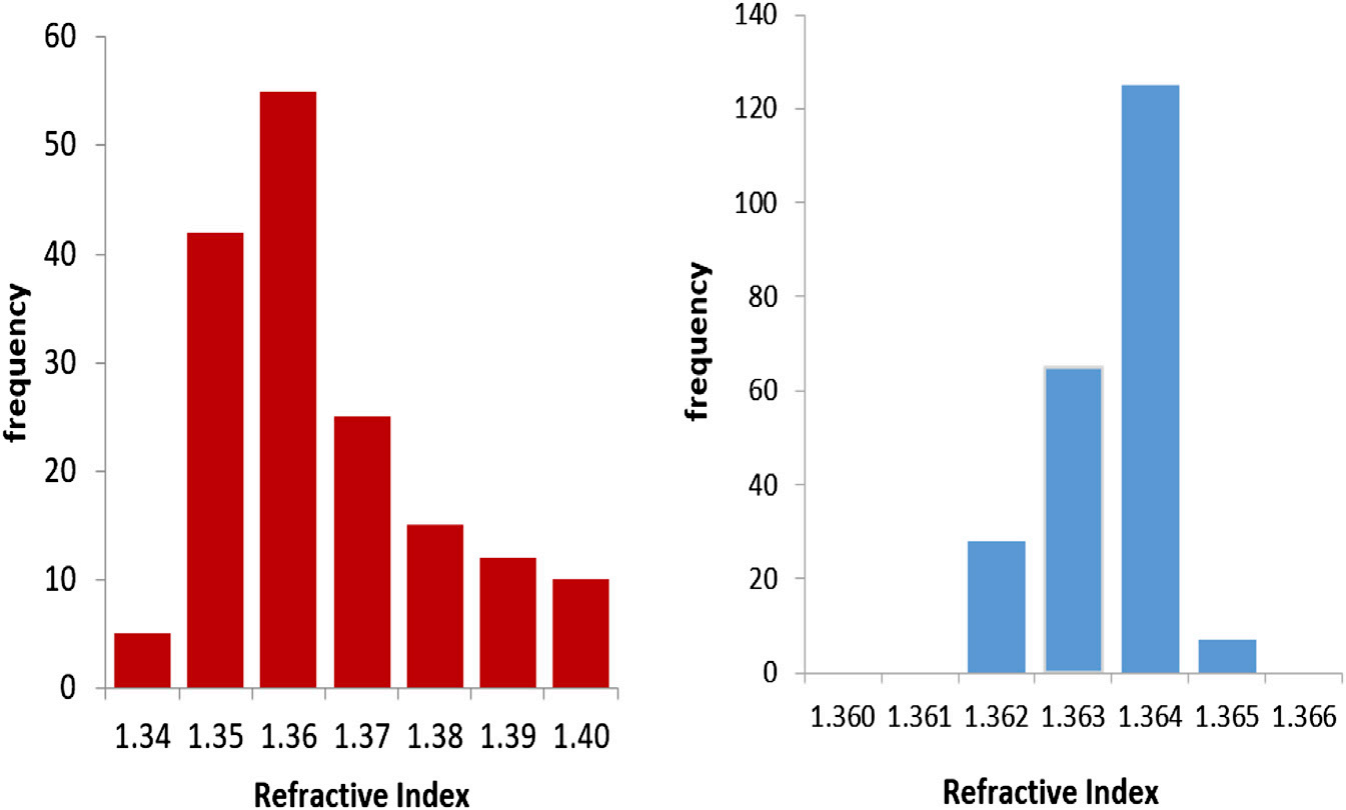
(*Left panel*) Frequency histogram representing the calculated average refractive index of fibroblasts (*n* = 164). (*Right panel*) Frequency histogram representing the refractive index acquired from measurements of one cell (*n* = 225). Note the intracell variation is much smaller than the intercell variation. To see this figure in color, go online.

While the measurements for the polystyrene beads show that the phase shift measurements acquired from the calculated phase images have a high accuracy, cells are not nearly as homogenous. Therefore, while intracell measurements were very consistent, there was a larger variation in intercell measurements of the phase shift and thickness (see Fig. 8). This highlights a limitation to the technique in that, as with all cell experiments of this kind, there is the potential for culture conditions to vary during imaging and/or transfer between microscopes, which could influence parameters tested. This would not be a problem if both measurements could be taken simultaneously, or if it could be guaranteed that the different measurements of phase shift and thickness were applied to the same cell in the same state. Regrettably, with the procedures used here, this is not possible. So far then, we are restricted to calculating average values over many different cells, and using these mean values to calculate the mean refractive index. The legitimacy of this method, in essence, relies on the cells displaying an acceptable degree of homogeneity. Because the 95% confidence limits of phase and thickness resulted in refractive index error values that presented at the third decimal place, it can be assumed that the cells are sufficiently homogenous for average values to be suitable for use in these calculations.

## Conclusions

It has been shown in this report that keratocytes change their refractive index upon their differentiation to fibroblasts. These changes will most likely result in a large increase in scattering per cell upon activation, and the increase in cell proliferation that accompanies the wound-healing response could explain the haze effect that has been documented in the weeks following some refractive surgical procedures.

## Author Contributions

S.J.G. performed research and wrote the article; K.M.M. designed research and edited the article; N.W. assisted with microscopy and edited the article; J.A. and C.S.K.-L. assisted with cell culture and edited the article; and C.K. assisted with theoretical modeling and edited the article.

## Figures and Tables

**Table 1:** Summary of results for phase shift and thickness and the resulting refractive index

Cell Type	Phase Shift ± SE (Radians)	Thickness ± SD (*μ*m)	Refractive Index ± SE
Keratocytes	4.04 ± 0.16 (*n* = 118)	7.2 ± 0.5 (*n* = 41)	1.381 ± 0.004
Fibroblasts	3.10 ± 0.09 (*n* = 164)	9.5 ± 0.3 (*n* = 28)	1.365 ± 0.003
